# Platelets derived citrullinated proteins and microparticles are potential autoantibodies ACPA targets in RA patients

**DOI:** 10.3389/fimmu.2023.1084283

**Published:** 2023-01-24

**Authors:** Minjie Xu, Rong Du, Wenping Xing, Xueting Chen, Jian Wan, Shengqing Wang, Li Xiong, Kutty Selva Nandakumar, Rikard Holmdahl, Hui Geng

**Affiliations:** ^1^ Hubei Key Laboratory of Genetic Regulation and Integrative Biology, School of Life Sciences, Central China Normal University, Wuhan, China; ^2^ Department of Rheumatology, Union Hospital, Tongji Medical College, Huazhong University of Science and Technology, Wuhan, China; ^3^ Department of Dermatology, Hospital affiliated to Central China Normal University, Wuhan, China; ^4^ Department of Environmental and Biosciences, School of Business, Innovation and Sustainability, Halmstad University, Halmstad, Sweden; ^5^ Division of Medical Inflammation Research, Department of Medical Biochemistry and Biophysics, Karolinska Institute, Stockholm, Sweden

**Keywords:** rheumatoid arthritis, citrullination, platelets, anti-citrullinated protein antibodies (ACPA), platelet derived microparticles (PDP)

## Abstract

Citrullinated neoepitopes have emerged as key triggers of autoantibodies anti-citrullinated protein antibodies (ACPA) synthesis in rheumatoid arthritis (RA) patients. Apart from their critical role in homeostasis and thrombosis, platelets have a significant contribution to inflammation as well. Although anuclear in nature, platelets have an intricate post-translational modification machinery. Till now, citrullination in platelets and its contribution to trigger autoantibodies ACPA production in RA is an unexplored research direction. Herein, we investigated the expression of peptidylarginine deiminase (PAD) enzymes and citrullinated proteins/peptides in the human platelets and platelet derived microparticles (PDP). Both PAD4 mRNA and protein, but not the other PAD isoforms, are detectable in the human platelets. With a strict filtering criterion,108 citrullination sites present on 76 proteins were identified in the human platelets, and 55 citrullinated modifications present on 37 different proteins were detected in the PDPs. Among them, some are well-known citrullinated autoantigens associated with RA. Citrullinated forms of thrombospondin-1, β-actin, and platelet factor-4 (also known as CXCL4) are highly immunogenic and bound by autoantibodies ACPA. Furthermore, ACPA from RA sera and synovial fluids recognized citrullinated proteins from platelets and significantly activated them as evidenced by P-selectin upregulation and sCD40 L secretion. These results clearly demonstrate the presence of citrullinated autoantigens in platelets and PDPs, thus could serve as potential targets of ACPA in RA.

## 1 Introduction

Accumulating evidences suggest citrullination is a pre-requisite for triggering autoantibodies ACPA generation in the susceptible rheumatoid arthritis (RA) patients (1–3). The presence of autoantibodies ACPA is a hallmark serological feature for classical RA, and has an association with a more destructive disease course, although their functional role might be both pathogenic and protective depending on their specificity (4). Interestingly, autoantibodies ACPA can be detected decades before the onset of clinical arthritis (5, 6) and it was reported that epitope spreading with an increased recognition of citrullinated antigens occurs before the onset of RA (7). B cells with citrulline specificity are likely to be triggered in response to citrullinated protein targets *in vivo* but the identity and role of these targets remain unclear.

Previous studies addressed autoantibodies ACPA targets present in the synovium and synovial fluid (8), cartilage (9), lungs (10), and inflammatory cells such as neutrophils and macrophages (11, 12). Herein, we investigated the expression of citrullinated proteins within the platelets and PDPs. Platelets are abundant hematopoietic cells present in the blood circulation. In addition to the well-described homeostasis and thrombosis functions, platelets have important immunologic activities too, by expressing multiple pattern recognition receptors, MHC molecules, and immune co-stimulatory molecules (13). Platelets are the major source of microparticles in the blood circulation, which enhances their communication within the immune system, and also involved in the crosstalk between immune and coagulation systems (14, 15). In RA, platelets and PDPs were detected in both the blood and the synovial fluid samples (16, 17). Depletion of platelets led to reduced vascular leakage in the arthritic joints and attenuated inflammation in the animal models (18, 19). Interestingly, previous studies reported that autoantibodies ACPA contributes to platelet activation *via* FcγR-dependent pathways (20), platelet-derived microparticals display autoantigen which could be recognition by ACPA, and result in perpetuating formation of inflammatory immune complexes (17).

Although platelets lack nuclei, they have an intricate post-translational modification (PTM) processing machinery. Based on a high resolution proteomic analysis, a diverse set of PTMs such as acetylation/deacetylation (21, 22), phosphorylation, arginylation (23), palmitoylation (24), glycosylation, and methylation (25) were described to fine-tune the biogenesis and function of platelets. Compared to these PTMs, citrullination processes occurring within the platelets have been less explored, including their potential contribution to trigger autoantibodies ACPA production in RA.

Here, for the first time, we provide strong evidences for the presence of citrullination proteome in platelets and PDPs. In addition, we identified certain citrullinated proteins as ACPA targeted autoantigens and in turn, ACPA promoted the activation of platelets. Our findings suggest an important role for citrullinated autoantigens present in the platelets and PDPs in RA pathogenesis.

## 2 Materials and methods

### 2.1 Patients and controls

Non-paired blood (n=40 for RA, n=11 for OA) and synovial fluid (SF, n=11 for RA, n=8 for OA) samples were collected from RA and OA patients attending the department of rheumatology, Union hospital, Tongji medical college, university of Wuhan, China. RA patients enrolled met the 1987 ACR/ELAR criteria (26). Blood samples from the age- and gender-matched healthy controls (n=40) were obtained from the blood bank center, University of Wuhan. SF was collected from RA and OA patients requiring arthrocentesis for their affected knees. This study was approved by the local ethics committee of Union hospital, Tongji medical college (No. [2020] IEC-J (130)), and all the study participants gave a written informed consent to participate in this project.

### 2.2 Isolation of platelets and monocytes from blood

Peripheral blood from the healthy volunteers (n=4) was collected into acid citrate dextrose tube and centrifuged at 200 g for 15 min to collect the platelet-rich plasma, to avoid contamination with other blood cells. Only the upper third of the platelet-rich plasma was used for analysis. Platelets were pelleted at 600 g for 10 min, and washed twice to reduce the contamination of serum proteins using HEPES-buffered Tyrode’s solution (5 mM HEPES, 145 mM NaCl, 5 mM KCl, 0.5 mM Na_2_HPO_4_, 1 mM MgSO_4_, and 5 mM glucose, pH 7.2). The purity of the collected platelets was determined by CD41-staining and subsequent flow cytometry analysis. For monocyte isolation, 5 ml heparinized blood was diluted 1:1 with PBS containing 0.46% sodium citrate and layered over a 5 ml Ficoll cushion, and then centrifuged at 400 g for 15 min. PBMC containing interphase was collected and washed twice with PBS-citrate solution. Thereafter, the PBMCs were cultured in RPMI-1640 medium containing 10% FBS, in six well culture plates for 1h, allowing monocytes to adhere to the plastics. The non-adherent lymphocytes were removed and the adherent monocytes were harvested for further use.

### 2.3 Identification of PAD isoenzymes expression by RT-PCR

RNA from the platelets and monocytes was isolated using RNAsimple Total RNA Kit (TIANGEN, Beijing, China). The cDNA was synthetized from 1 µg of RNA using HiScript III 1st Strand cDNA Synthesis Kit (Vazyme, Nanjing, China). For PCR, 1 μl of cDNA was added to 25 μl of 2 × Phanta Flash Master Mix (Vazyme, Nanjing, China) containing Phanta Flash Super-Fidelity DNA Polymerase and, 0.5 mM forward and reverse primers. PCR was performed using a ETC811 thermocycler: 30 s at 98°C, 30 cycles of 10 s, 98°C; 5 s, 50-55°C; 5 s, 72°C, and one min elongation time at 72°C. 5 μl of the PCR product was analyzed by electrophoresis on a 1.5% agarose gel. The primers used to amplify the PAD isoforms and β-actin are presented in **Table S1**.

### 2.4 Isolation of the PDPs

For the collection of platelet derived particles (PDPs), platelets were resuspended at 2 × 10^8^/ml and stimulated with 5 μM of ADP (Sigma-Aldrich, Shanghai, China) in platelet aggregometer for 5 min at 37°C. Platelet activation was terminated by placing the tube on ice and then adding ice-cold 0.1% v/v termination buffer containing 10% NP-40, 20 mM PMSF, 200 g/mL trypsin inhibitor, 50 mM N-ethylmaleimide, 10 mM EDTA, 1% SDS and 100 mM benzamidine. Intact platelets and the platelet clots were carefully removed by centrifuging at 800 g for 10 min, sequentially for three times, and the supernatant was harvested each time and pooled before the final spin, which yielded the PDPs.

### 2.5 MS analysis

Platelet pellets and PDPs were lysed in 8 M or 2 M urea solution, respectively. To this lysate, 10 mM HEPES (pH 8) was added and sonicated at 4°C for 20 min. One fraction of platelet sample was separated by gel electrophoresis and subjected to in-gel trypsin digestion as described earlier (27). Briefly, gel slices (20 slices/lane) were excised and the proteins were reduced with 64 mM of dithiothreitol (DTT) at 56°C for 60 min, followed by an alkylation step in the dark for 45 min with 130 mM iodacetamide (IAA). Proteins were digested with trypsin/Lys C mixture (Promega, Wisconsin, USA) overnight at 37°C. The peptides were purified with acetonitrile/0.1% aqueous formic acid 1:1 (v/v), and re-solubilized in 0.1% aqueous formic acid prior to LC-MS/MS analysis. Another fraction of platelet sample as well as PDPs were prepared using a filter-aided sample preparation protocol (FASP) (28). Each sample (100 μg) was transferred to 10 kDa cutoff spin-filter (Millipore, Ireland, UK), reduced with DTT, blocked by IAA and digested with trypsin/Lys C mixture for overnight at 37°C. The peptides were eluted from the spin filters, acidified with 0.1% formic acid, and then desalted with a C18 pipet tip (Millipore, Ireland, UK) for further use.

Peptides were loaded onto a Thermo Acclaim Pepmap precolumn followed by an Acclaim Pepmap Easyspray analytical column. Separation was performed using a Thermo Easy-nLC 1200 at a flow rate of 300 nL/min with a gradient of 2-35% organic phase (0.1% formic acid in acetonitrile) over 200 min. Peptides were analyzed using a high-energy collisional dissociation (HCD) fragmentation mode by data-dependent MS/MS acquisition, with the following settings: MS1-scan resolution 70 ;000, the AGC target was 3e6; MS2-scan resolution 17 ;500, the AGC target was 5e4; and the maximum injection time was 50 ms, NCE stepped set 27. The scan range was set at a resolution of 350-2,000 m/z and the 20 most intense precursors were chosen by data-dependent mode.

### 2.6 Protein identification and quantification

MaxQuant software (version 1.6.6.0) was used for the identification of peptides and proteins against the human proteome database. Peptide identification was performed with a mass tolerance up to 5 ppm for precursor ion and MS/MS tolerance to 20 ppm. A false discovery rate (FDR) was set to 0.01 at the peptide and protein level. Relative protein quantities were calculated by summing the unique peptide peak areas of each protein in MaxQuant using the LFQ (label-free quantitation) feature (29).

### 2.7 Identification of citrullination

To ensure the accuracy of identifying citrullination, we applied a strict filtering criterion to verify the citrullinated side chains, as previously described (30, 31). At first, the precursor ions were extracted with a mass tolerance up to 5 ppm to prevent the forced ^13^C mis-assignment. All the total peptide spectral matches (PSMs) in the C-terminal citrullination site were removed due to inability of trypsin/Lys C catalytic activity to citrulline residues. Next, the assigned citrullinated MS2 spectra were manually inspected to exclude 0.984 Dalton mass increments occurring from deamidation with the neighboring N/Q residues. Only the complete fragment ion series (b, y ions) covering the citrullinated sites were considering as correct assignment. Identification of citrullinated sites was further confirmed by the occurrence of neutral loss on the precursor or fragment ions and a shift in the delayed retention time.

### 2.8 *In vitro* citrullination

Human recombinant thrombospondin-1 (TSP-1, Sigma-Aldrich), beta-actin (β-actin, Genway, San Diego, USA), or platelet factor 4 (PF4, Sigma-Aldrich) was incubated with PAD4 enzyme (Sigma-Aldrich) in the siliconized tubes containing 100 mM of Tris pH 7.7, 1 mM of DTT, and 5 mM of CaCl_2_. After 24 h at 37°C, reactions were stopped by adding 0.1% of trifluoroacetic acid. Citrullinated and non-citrullinated samples were loaded on SDS-PAGE followed by in-gel digestion for further mass spectrometry analysis of citrullination sites, as well as to screen for binding of ACPA by ELISA.

### 2.9 ELISA for autoantibodies ACPA measurement

The plates were coated with 200~500 ng/well of citrullinated or non-citrullinated forms of TSP-1, β-actin, and PF4 in 0.1 M NaHCO_3_ (pH 9.6), overnight at 4˚C. The plates were washed with PBS/Tween 0.05%, blocked with 1.5% BSA/PBS for 1 h, at RT, and incubated with RA sera (1:100 dilution) for 2 h, RT. Then the plates were washed and developed with HRP-conjugated rabbit anti-human IgG polyclonal antibodies for 1 h. TMB substrate was added and incubated at 37°C for 5~10 min and the reaction was stopped with 1.0 M sulfuric acid (H_2_SO_4_). The absorbance (A450 nm) was read using a microplate reader (Thermo, Shanghai, China). The absorbance value of the pooled healthy sera was set at an arbitrary unit (AU) value of 1 and was used as a standard for all other tests (1 AU/μl).

### 2.10 Affinity purification of autoantibodies ACPA

Autoantibodies ACPA were purified from selected RA patients’ synovial fluid and serum samples having anti-CCP2 IgG level > 500 AU/ml, using Protein G beads and CCP2-coated resins. CCP2 peptide were purchased from Euro Diagnostica (Malmö, Sweden, obtained from Biolead China), and couple to CNBr-activated Sepharase 4B beads (GE Healthcare) according to the manufacturer’s protocol. SF (5~8 mL/sample) was centrifuged at 4,000 rpm for 10 min, and the supernatant collected was treated with hyaluronidase to decrease the viscosity of the samples. Proteins were precipitated by using a saturated ammonium sulfate solution, and then dissolved in PBS and dialyzed extensively against PBS. While the serum samples were centrifuged and diluted 1:5 in PBS before purification. At first, total IgG was enriched from SF and serum samples using protein G beads. To purify ACPA, 1 mg/ml of CCP2 peptide was coupled to 2 ml of NeutrAvidin Plus UltraLink resin (Pierce Biotechnology/Thermo Scientific) for 1 h at RT. Purified total IgG from SF and serum samples was then applied to the CCP2-coated resin, and washed with PBS. ACPA were eluted by adding 0.1 M glycine-HCl buffer (pH 2.7), neutralized with 1 M Tris (pH 9) and then dialyzed against PBS.

### 2.11 Platelet activation and flow cytometry analysis

To block the binding of IgG-Fc with CD32, platelets (2×10^8^/ml) were first pre-incubated with 20 μg/ml anti-human CD32 (6C4, eBioscience, Rockford, IL) for 1 h at 37°C and washed twice with PBS, To determine the capability of ACPA mediated platelets activation, platelets were incubated with seleted ACPA ^high^ sera, or SF (1:20 dilution) from RA patient or purified autoantibodies ACPA (10 μg/ml in PBS + 0.1% BSA) at 37°C for 90 min. Then supernatants were collected by centrifugation (400×g, 15 min, 20°C) and platelets were resuspended for flow cytometric analysis. The activation status of platelets was determined by flow cytometry by measuring the expression of CD62P and sCD40L level in the collected supernatants. Platelets were incubated with anti-CD62P-FITC (clone AK-4; BD Biosciences, New York, USA) which was diluted in HEPES-buffered Tyrode’s solution, pH 7.2 (1:50 dilution) for 30 minutes at RT, after which cells were fixed using 1% paraformaldehyde and analysed by NovoCyte Flow Cytometer and NovoExpress software (Agilent, Palo Alto, USA). The sCD40L level was determined by using an ELISA Kit (eBioscience, San Diego, USA).

### 2.12 Statistical analyses

For normally distributed populations, mean ± SD values were used and Student’s t test was applied to evaluate the statistical difference between the groups. Differences in antibody levels were examined using the non-parametric Mann-Whitney U test. Data were analyzed using GraphPad Prism version 9.0. The p values < 0.05 were considered as statistically significant.

## 3 Results

### 3.1 PAD4 expression was detectable in the human platelets

**Figure 1:**
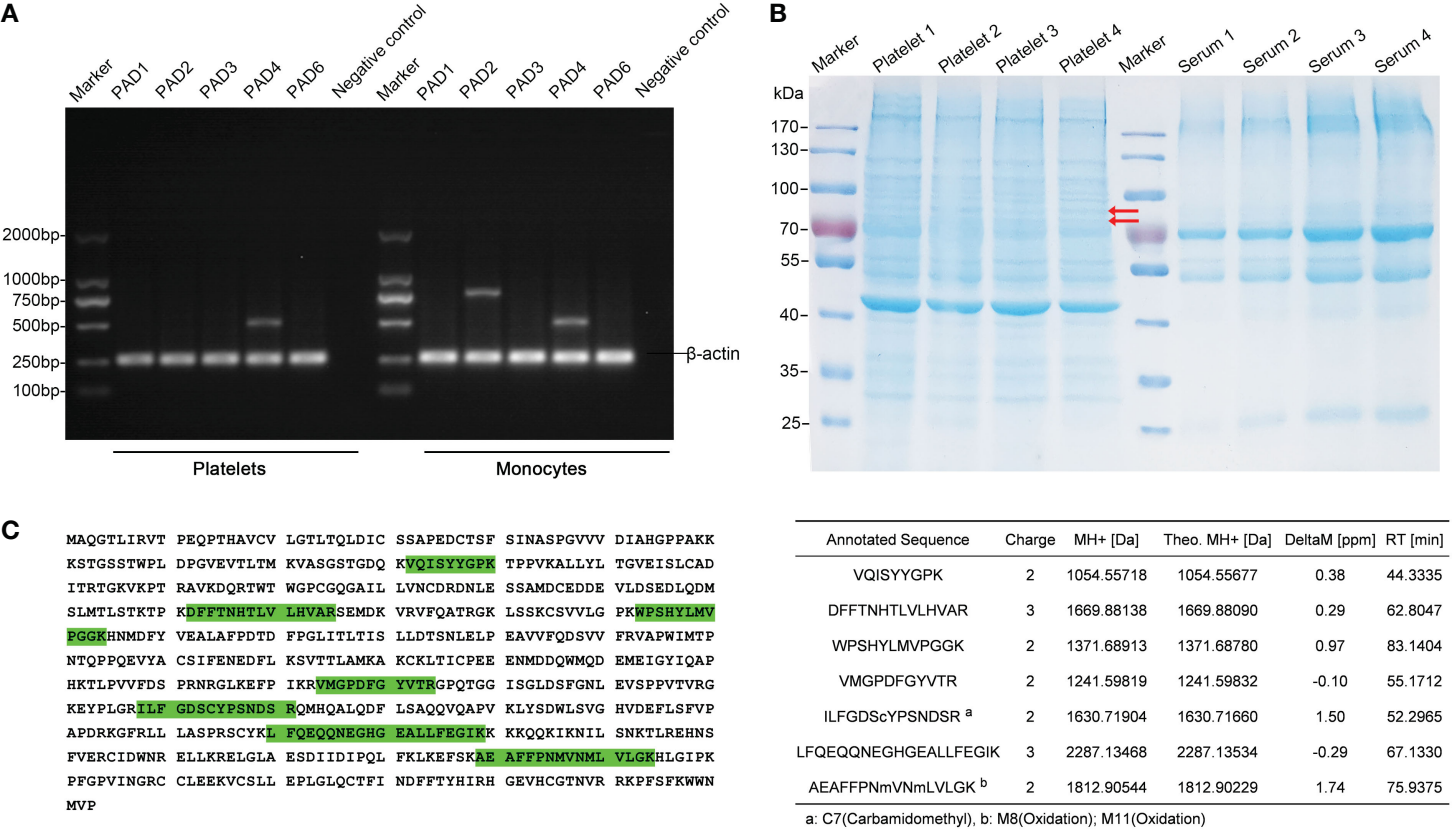
Identification of PAD4 expression in the human platelets. **(A)** Agarose gel electrophoresis of RT-PCR products after amplifying the mRNA of PAD isoforms in platelets and monocytes. The β-actin expression was used as an internal control. PCR without the template served as a negative control. **(B)** SDS-PAGE depicting the proteins from platelets. Lanes 2-5 are corresponding to platelet samples divided into 20 slices from top to bottom, followed by an in gel-digestion and LC-MS/MS analysis. Arrow indicates PAD4 expression in the gel by MS analysis. Lanes 7-10, show the paired serum samples indicating the limitation present in the carry-through of plasma proteins into platelet proteomics. **(C)** Detected PAD4 peptide sequence coverage. Left section: the identified tryptic peptides are indicated in green shadow. Right section: list of detected and mapped peptides.

To date, five Ca^2+^-dependent PAD isozymes designated as PAD1-4, and PAD6, which catalyze the conversion of arginine to citrulline have been identified, each with a unique tissue and cell distribution pattern (32). To examine the presence of PAD isoforms in human platelets, we first investigated the mRNA expression of all the five PAD isozymes by RT-PCR. Platelets (> 98% CD41^+^ cells by flow cytometry analysis, data not shown) and paired monocytes were isolated from the peripheral blood of four healthy volunteers. Total RNA was isolated from the platelets and monocytes, and each isotype of PAD gene was amplified by RT-PCR. Surprisingly, a substantial number of PAD4 mRNA was detectable in all the four platelet samples, but not the mRNAs of PAD2 or PAD1, 3 and 6 isozymes, the other isoforms described to be present in the platelets. On the other hand, we detected both PAD2 and PAD4 mRNAs in the monocytes (**Figure 1A**).

We then analyzed the expression of PAD proteins by mass spectrometry (MS). To make the platelet samples less complex to achieve a higher resolution of PADs, platelet proteins were fractioned by gel electrophoresis into 20 gel slices prior to MS analysis (**Figure 1B**). Clearly, unique peptides from PAD4 could be detected in two gel slices between 70 to 100 KDa bands in all the four platelet samples, which correspond to the expected molecular mass of PAD4 enzyme. BLAST search in the UniProt confirmed the aa sequences of these peptides to be unique for human PAD4 isoform, with an amino acid sequence coverage of 14.5%. In contrast, peptides of PAD2, or PAD1, 3, and 6 isoforms were not detectable in the platelet protein extracts (**Figure 1C**). Taken together, the identified PAD4 mRNA and protein expressed in the platelets suggests the importance and critical contribution of PAD4 isoform in citrullinating the proteins/peptides within the platelets in humans.

### 3.2 Identification of citrullinated proteins in human platelets

Next, we identified the proteins and specific arginine residues that were citrullinated in the platelets. To facilitate the peptide recovery, we performed a filter-aided sample preparation (FASP) method in the four platelet samples. MS analysis identified more than 44, 639 unique peptides and 3, 972 proteins with an abundance covering 10 orders of magnitude. The 100 most abundant proteins are well-known cytoskeleton, α-granule, and cytoskeletal-linked signaling proteins (**Table S2** and **Figure S1**).

**Figure 2:**
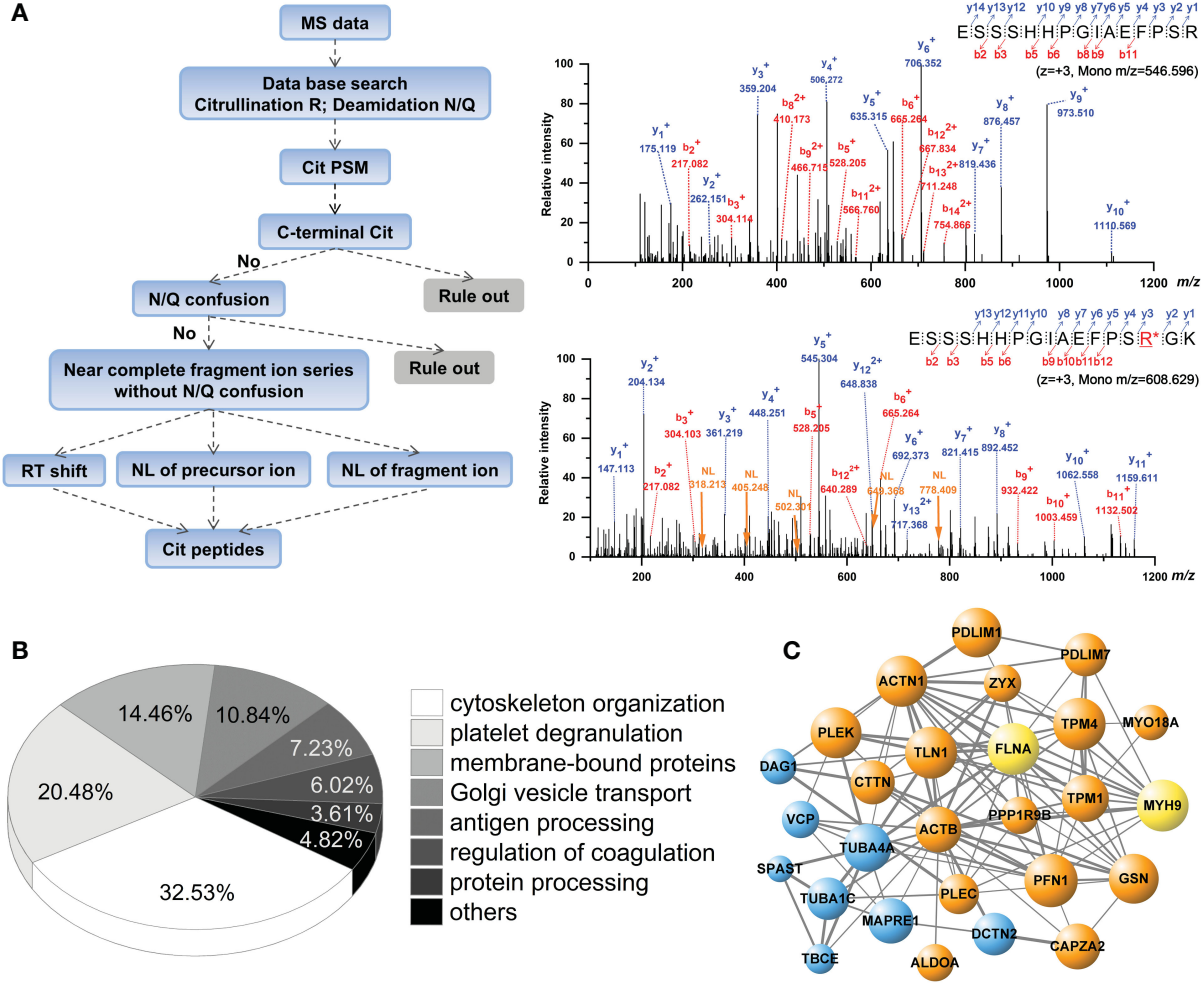
Analysis of citrullinated peptides present in the human platelets by mass spectrometry. **(A)** Decision tree for the identification of citrullination is shown in the left panel. Representative dimer-isotopic MS2 spectrum of non-citrullinated and citrullinated 560E–575K peptide of human fibrinogen α is shown in the right panel. Purple arrow indicates the neutral loss fragment (NL, -43.0058 Da) corresponding to y3+~y7+ ions. **(B)** Pie-chart showing the classification of identified citrullinated proteins in the platelets, according to functional categories. **(C)** Protein interaction network of citrullinated proteins. Orange bobbles show the actin-myosin filaments; blue bobbles show the microtubules; yellow bobbles show the actin-myosin filaments and microtubules.

When peptides were analyzed for citrullinated modifications, a strict filtering criterion was applied to eliminate the interferences from N/Q deamidation and ^13^C isotopes (**Figure 2A**). Each MS/MS spectrum was manually verified by comparing it with the theoretically fragmented ions. The FASP short-gun MS and SDS-pre-fraction LC-MS/MS analysis revealed 108 citrullination sites on 76 different proteins (**Table 1**). These 76 citrullinated proteins included previously characterized substrates of PAD enzymes, such as actin, alpha-enolase, fibrinogen α/β, filamin-A, transgelin-2, and tubulin α (11, 30). In addition, some proteins with more specific roles in platelet functions were observed to be modified by citrullination, including integrin α-IIb, plasminogen, platelet glycoprotein Ib α, platelet glycoprotein IX, TSP-1, and von Willebrand factor. We noted that proteins with a broad range of expression levels were modified by citrullination, including highly and less abundant proteins in human platelets (**Table 1**).

**Table 1:** Citrullination modifications present in human platelets.

Accession	Description	Site	iBAQ	Accession	Description	Site	iBAQ
P12814	Alpha-actinin-1	R350	2	P35579	Myosin-9	R1912	23
		R760				22		R1923
Q08043	Alpha-actinin-3	R74	P23368	ME2	R413	422
P06733	Alpha-enolase	R429	3660	Q96SB3	Neurabin-2	R810	1518
P08758	Annexin A5	R18	55	O00151	PDLIM1	R177	51
P60709	Beta-Actin	R256	444	Q9NR12	PDLIM7	R137	304
Q9BXS5	AP1M1	R304	580			R158	
O95810	CAVIN2	R141	128	P08567	Pleckstrin	R307	18
P14209	CD99 antigen	R170	432	Q15149	Plectin	R2767	626
Q96G23	Ceramide synthase	R363	1038			R3337	
Q00610	Clathrin	R574	190	P07737	Profilin-1	R75	4
Q6PJW8	Consortin	R250	384	P02760	Protein AMBP	R185	1177
Q9H7D0	DOCK5	R1721	2063	Q9H0Q0	Protein FAM49A	R147	1042
Q13561	Dynactin subunit	R212	351	Q15404	RSU1	R165	33
O00429	DNM1L	R108	218	Q9H0U4	RAB1B	R183	186
Q14118	Dystroglycan	R689	1437	Q9NS28	RGS18	R205	516
Q9NZN3	EHD3	R404	98	O94804	STK10	R451	1577
P11021	HSPA5	R49	109	P02768	Serum albumin	R281	11
Q9BSJ8	ESYT1	R1003	366			R509	
P47755	CAPZA2	R109	245	O75368	SH3BGRL	R58	198
P02671	Fibrinogen alpha	R123	32	Q9UJC5	SH3BGRL2	R74	165
		R271		Q9UBP0	Spastin	R488	3193
		R287		Q14247	CTTN	R59	259
		R510				R434	
		R573		Q7Z422	SZRD1	R65	2288
		R591		O00161	SNAP23	R148	140
P02675	Fibrinogen beta	R60	28	Q9Y490	Talin-1	R35	27
P21333	Filamin-A	R2001	15			R181	
		R2003				R606	
		R2334				R2154	
		R2484		P50991	CCT4	R153	393
P04075	ALDOA	R331	48	O43396	TXNL1	R211	447
O15117	FYB1	R19	288	P07996	Thrombospondin-1	R314	25
Q3ZCW2	LGALSL	R106	122			R412	
P06396	Gelsolin	R397	39			R751	
Q02153	GUCY1B1	R197	979			R767	
P0DMV9	HSPA1B	R357	234			R926	
P10316	HLA-A	R86	3871	Q01664	TFAP4	R293	3887
Q9H2U2	PPA2	R84	959	P37802	Transgelin-2	R160	20
P23229	Integrin alpha-6	R635	292	P55072	VCP	R210	134
P08514	Integrin alpha-IIb	R104	42	P09493	TPM1	R55	84
		R334		P67936	TPM4	R27	10
		R399		Q9BQE3	Tubulin alpha-1C	R422	371
		R433		P68366	Tubulin alpha-4A	R79	80
		R799		Q15813	TBCE	R13	2743
P05106	Integrin beta-3	R505	35	O43399	TPD52L2	R182	382
		R662		Q92614	MYO18A	R1302	1524
P13645	KRT10	R15	967	P46459	NSF	R533	1014
P35908	KRT2A	R524	1917	P18206	Vinculin	R547	49
Q15691	MAPRE1	R162	171			R622	
Q13201	Multimerin-1	R450	160			R874	
P35579	Myosin-9	R165	23	P04275	von Willebrand factor	R1136	214
		R930				R1597	
		R1342		Q15942	Zyxin	R517	70

String and GO analysis showed 32.53% (n = 27 proteins) of the identified citrullinated proteins are involved in the organization of cytoskeleton (p = 3.21×10^−11^) related to actin-myosin filaments and microtubules in platelets (**Figures 2B, C**). Protein interactome analysis indicated that a substantial portion of the citrullinated proteins was enriched after platelet degranulation (20.48%, p = 5.80×10^−16^; **Figure 2B**). Previous studies using brain, gut mucosa, and myocardial tissues showed a more preferential targeting of extracellular- and membrane-bound proteins by citrullination (33–35). Similarly, in our analysis, proteins annotated as membrane-bound proteins involved in cell-cell and cell-substrate adhesions were over-represented in the human platelet citrullinome (14.46%, p = 8.57×10^−10^), especially in the processes involved in integrin α-IIb and fibrinogen, as well as in the interactions related to the cytoskeletal remodeling and platelet degranulation.

### 3.3 Citrullinated proteins are released in the platelet derived microparticles

Since human platelets expressed PAD4 enzyme and abundant citrullinated proteins were identified, we next addressed whether platelets could release the citrullinated proteins outside the cells. To investigate the citrullinated protein contents of PDPs, platelets from 12 volunteers were stimulated with ADP to induce the release of PDPs, and then the PDPs were harvested and subjected to MS analysis. We identified 5, 476 peptides of 687 proteins at FDR 0.01 (**Table S3**). As expected, many PDPs were found to be citrullinated, with the identification of 55 citrullinated peptides from 37 different proteins (**Table 2**). As described above, some proteins having more specific roles in platelet functions were observed to be citrullinated in the PDPs as well, such as integrin α-IIb, multimerin-1, pleckstrin, TSP-1, and von Willebrand factor. However, the majority of proteins identified to be in the citrullinated form were present within a single sample. This may be due to technical reasons, such as varying sampling depth or low abundance of the modification, or individual differences in the citrullination patterns.

**Table 2:** Citrullination modifications detected in platelet delivery PRMs.

Accession	Description	Site	iBAQ	Accession	Description	Site	iBAQ
P35609	Alpha-actinin-3	R067	620	P02776	Platelet factor 4	R080	1
P02749	Beta-2-glycoprotein 1	R210	122	P07359	PG-Ib-alpha	R080	74
P60709	β-actin	R256	4	P01127	PDGFB	R142	110
P27797	Calreticulin	R177	176	P08567	Pleckstrin	R307	21
O95810	CAVIN2	R141	220	P07737	Profilin-1	R075	13
P10909	Clusterin	R194	19	A6NCN2	KRT87P	R072	559
P01024	Complement C3	R1591	69	P02787	Serotransferrin	R239	25
P29279	CCN2	R240	161			R651	
Q86UX7	FERMT3	R098	48			R682	
P02671	Fibrinogen alpha	R218	15	P02768	Serum albumin	R122	3
P02675	Fibrinogen beta	R410	14			R141	
P21333	Filamin-A	R2003	64			R281	
P06396	Gelsolin	R048	23			R469	
		R295				R509	
		R397		Q9H299	SH3BGRL3	R047	164
P04406	GAPDH	R080	32	P09486	SPARC	R181	5
P01857	IGHG1	R299	7	Q9Y490	Talin-1	R606	80
P08514	Integrin alpha-IIb	R312	136	P07996	Thrombospondin-1	R314	6
P35527	KRT9	R327	153			R412	
Q14766	LTBP1	R1445	85			R460	
		R1508				R751	
		R1538				R926	
Q13201	Multimerin-1	R450	65	P01137	TGF beta-1	R255	67
		R589		P67936	Tropomyosin alpha-4	R065	17
		R890		P07437	Tubulin beta	R086	44
P14543	Nidogen-1	R1017	54	P04275	von Willebrand factor	R647	102
P02775	Platelet basic protein	R062	2			R1597	
P02776	Platelet factor 4	R051	1				

### 3.4 Autoantibodies ACPA bind to citrullinated TSP-1, β-actin, and PF4

To address the citrullination activities by PAD4 on platelet proteins, we chose TSP-1, β-actin, and PF4 as testing substrates, because of their identified citrullinated forms present in the platelets and PDPs. After the treatment with PAD4 *in vitro*, in total 18 arginine residues of rTSP-1 were citrullinated, which included the five *in vivo* citrullination sites of TSP-1 observed in the platelets and PDPs, as well as additional 12 citrullinated residues (**Table 3**). For β-actin samples treated with the PAD4 enzyme, 12 citrullination sites were identified, including the 10 citrullination sites that had previously been reported by another group (36) (**Table 3**). Human PF4 (also named as CXCL4) contained 4 Arg residues, including Arg51 and Arg80 that could be citrullinated *in vitro* (**Table 3**). Over all, PAD4 was efficient in citrullinating the platelet proteins TSP-1, β-actin, and PF4.

**Table 3:** Citrullination sites present in TSP-1, β-actin, and PF4 by PAD4 catalysis.

protein	site	Annotated Sequence^a^	*In vivo*
TSP-1 (18)	R83	FQDLVDAVRAEK	
	R101	TRGTLLALERK	
	R196	DLASIARLRIAK	
	R216	GGVNDNFQGVLQNVRFVFGTTPEDILR	
	R285	DLQAICGISCDELSSMVLELRGLR	
	R288	GLRTIVTTLQDSIR	
	R314	RPPLCYHNGVQYR	R314
	R404	GRSCDSLNNR	
	R412	SCDSLNNRCEGSSVQTR	R412
	R421	CEGSSVQTRTCHIQECDK	
	R458	QDGGWSHWSPWSSCSVTCGDGVITRIR	
	R460	IRLCNSPSPQMNGK	
	R517	RSRLCNNPTPQFGGK	
	R607	EVPDACFNHNGEHRCENTDPGYNCLPCPPR	
	R675	CNYLGHYSDPMYRCECK	
	R751	IPDDRDNCPFHYNPAQYDYDR	R751
	R767	DNCPFHYNPAQYDYDRDDVGDR	R767
	R926	DSDGDGRGDACKDDFDHDSVPDIDDICPENVDISETDFR	R926
β-Actin (10)	R62	RGILTLK	
	R95	IWHHTFYNELRVAPEEHPVLLTEAPLNPK	
	R183	LDLAGRDLTDYLMK	
	R196	ILTERGYSFTTTAER	
	R206	GYSFTTTAEREIVR	
	R210	REIVRDIK	
	R254	SYELPDGQVITIGNERFR	
	R256	FRCPEALFQPSFLGMESCGIHETTFNSIMK	R256
	R312	DLYANTVLSGGTTMYPGIADRMQK	
	R372	QEYDESGPSIVHRK	
PF4 (2)	R51	TTSQVRPR	R51
	R80	AGPHCPTAQLIATLKNGR*K	R80

a red color indicates citrullinated arginine residue.

**Figure 3:**
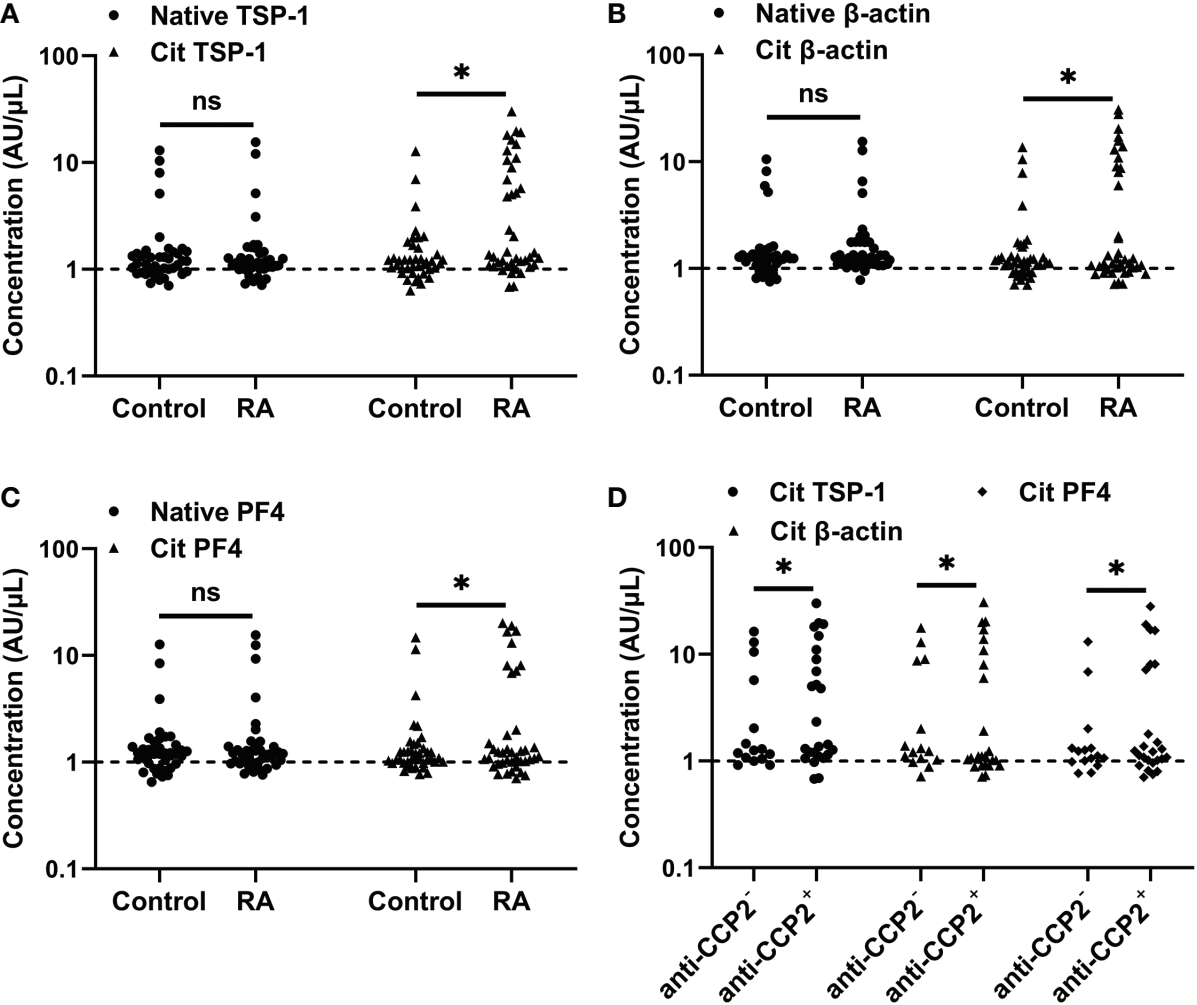
Measurement of citrullinated TSP-1, β-actin, and PF4 - specific antibodies in the RA sera. **(A-C)** Determination of citrullinated TSP-1, β-actin, and PF4-specific antibodies in the sera (1:100 dilution) of healthy controls and RA patients. **(D)** Distribution of citrullinated TSP-1, β-actin, and PF4-specific antibodies in the anti-CCP2^+^ and anti-CCP2^-^ RA sera. Broken lines indicate the cutoff values. Cit, denotes citrullination, *p < 0.05, ns = not significant.

We then investigated whether citrullinated TSP-1, β-actin, and PF4 could serve as autoantigens in RA. The reactivity of RA serum with native and citrullinated forms of TSP-1, β-actin, and PF4 was assessed by ELISA. RA serum showed a low reactivity towards native forms of TSP-1, β-actin, and PF4, similar to healthy controls (**Figures 3A–C**). In comparison, an increased frequency of RA sera showed reactivity to citrullinated forms of TSP-1 (37.5%), β-actin (30.0%), and PF4 (22.5%) (**Figures 3A–C**), while the control serum had no such increased reactivity. As expected, the majority of the citrullinated TSP-1, β-actin, and PF4 positive RA cases were confined to the anti-CCP2^+^ RA group (**Figure 3D**).

### 3.5 Autoantibodies ACPA bind to citrullinated platelet proteins and enhance the activation of platelets.

**Figure 4:**
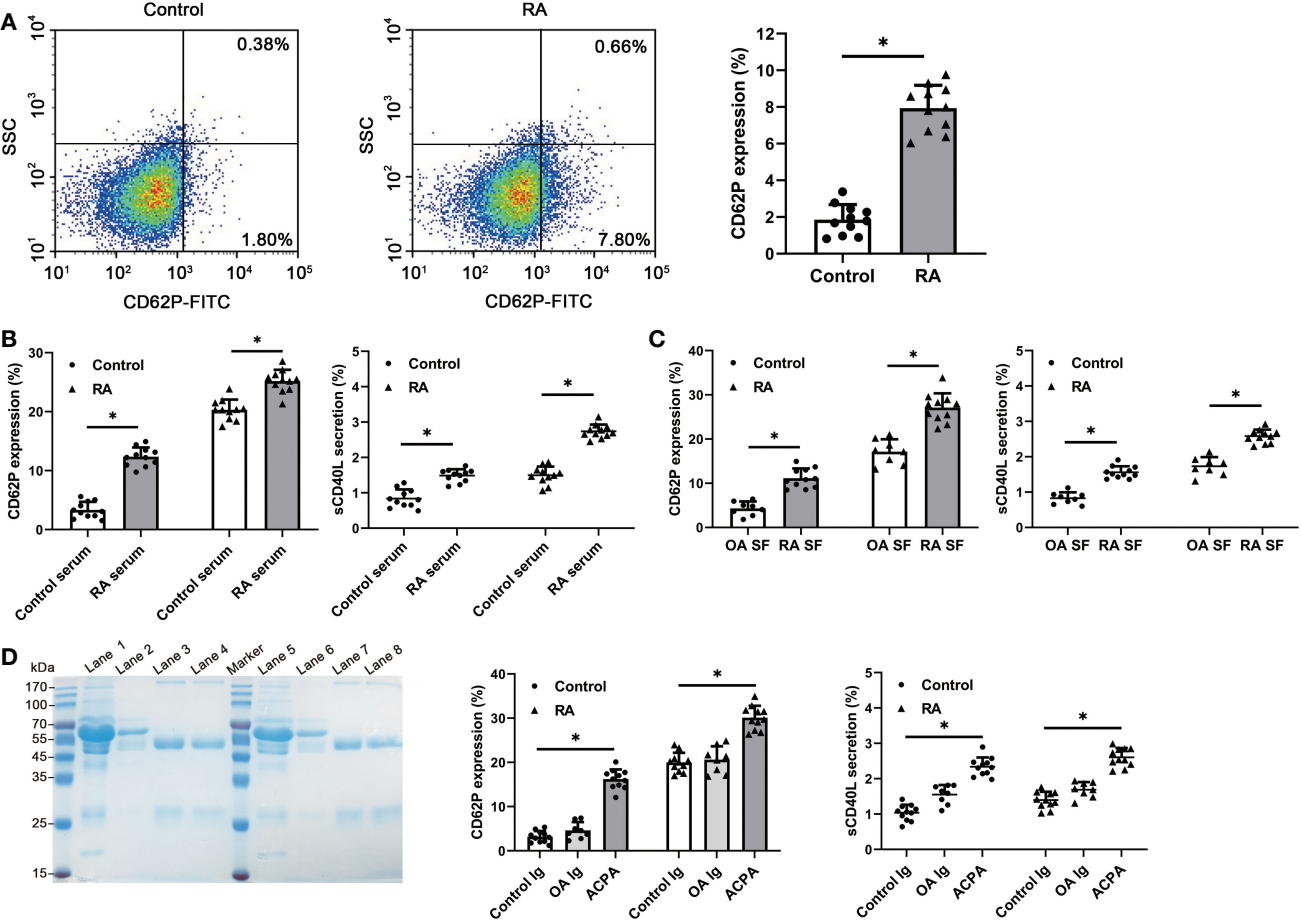
Detection of platelet activation after RA serum, SF or purified ACPA stimulation. **(A)** Flow cytometric quantification of CD62P expression in the platelets from healthy controls and RA patients. Data are representative of six independent experiments. **(B, C)** Both the serum and SF from RA induced significantly a higher level of CD62P expression and sCD40L secretion in RA and healthy platelets, compared to the healthy serum or OA SF (RA serum and SF: n=11, healthy control serum: n=11, OA SF: n=8). **(D)** Purified ACPA from RA SF and serum samples has significantly enhanced the CD62P expression and sCD40L secretion in RA and healthy platelets. SDS-PAGE showing the purified ACPA from SF of 2 RA patients by protein G plus beads and CCP2-peptide coated resin. Lane1/lane5: SF; Lane2/lane6: flow through from protein G column; Lane3/lane7: eluted IgG from protein G column; Lane4/lane8: eluted ACPA from CCP2-coated resin. Data are presented as mean ± SD, *p < 0.05.

Since citrullinated proteins are present in the human platelets, we hypothesized that autoantibodies ACPA in RA patients may react to the citrullinated platelet proteins and interact with the activation of platelets. To investigate the ACPA reactivity, we used RA plasma and SF having a high titer of anti-CCP2 antibodies (> 500 AU/ml) in the stimulation experiments. A higher basal activation status was detected in platelets from high anti-CCP2^+^ RA patients, as indicated by an increased expression of P-selectin (RA patients 7.94 ± 1.25% vs control 1.86 ± 0.82; p < 0.05) (**Figure 4A**). Upon stimulation, both the serum and SF from high anti-CCP2^+^ RA patients have potently induced platelets collected from healthy and RA patients, when compared to the healthy serum or OA SF, and also increased the production of sCD40L (**Figures 4B, C**). We further purified the ACPA from RA patients’ SF and serum samples. Pooled ACPA samples have significantly increased the expression of both P-selectin and the production of sCD40L in the healthy and RA neutrophils, when compared to control IgG or IgG from OA SF (**Figure 4D**). These observations demonstrate ACPA reactivity to the citrullinated proteins present in the platelets and their subsequent activation of platelets.

## 4 Discussion

**Figure 5:**
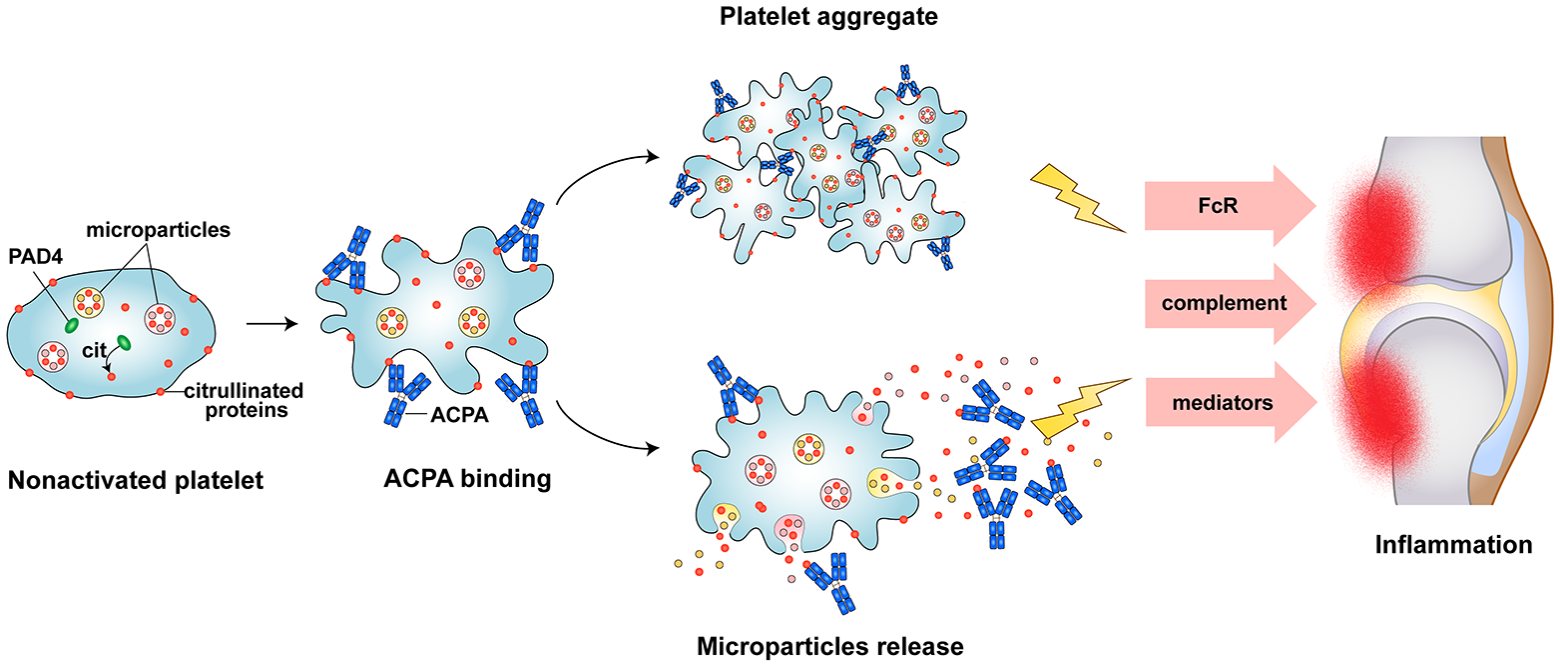
Schematic picture of platelet as a source of autoantigens and persistent inflammation in RA. Autoantibodies ACPA recognized citrullinated proteins in platelet, result in platelet activation, aggregation, and microparticles releasing. This will further lead to citrullinated proteins exposure to the immune cell, triggering of ACPA production and contribute to the persistent inflammation.

In this study for the first time, we have demonstrated the expression of PAD4 enzyme and citrullinated proteins present within the human platelets and PDPs. In addition, we also found platelet activation mediated by ACPA from RA patients. In RA, platelet aggregates are often observed in the blood and the joint, microparticles releasing as consequence of platelet activation accumulate in the joints of patients. While the mechanistic events that lead to platelet activation in RA have not been extensively characterised. We hypothesized that citrullinated proteins from platelet and PDP may play prominence role in stimulating platelet activation in RA. Here we shown that plenty of citrullinated protein present in platelets and PDPs can be recognized by autoantibodies ACPA. and ACPA can stimulate platelet activation lead to releasing active inflammatory molecules and citrullinated autoantigens that may sustain inflammatory responses in RA joint (**Figure 5**).

The most noticeable finding is the unique expression of PAD4 in platelets both at mRNA and protein levels, which citrullinated platelet proteins/peptides. Recently, PAD4 was identified to be inextricably associated with RA than other PAD isoforms. PAD4 enzymes have been founded in the rheumatoid synovial tissue and fluid (37, 38). In addition, PAD4 gene and certain SNPs were detected within a susceptibility locus associated with RA (39). PAD4 is unique because only this PAD isoform contains a canonical nuclear localization signal (NLS) found predominantly in the nucleus (40). Many *in vitro* experiments demonstrated that both PAD4 and PAD2 isoforms are efficient in generating citrullinated target sites for autoantibodies ACPA, while ACPA preferentially bind to histone H3 and fibrinogen citrullinated by PAD4 than PAD2 (41, 42).

Our data uncovered a list of previously unreported citrullinated proteins present in the platelets and PDPs. We also identified citrullinated forms of fibrinogen α/β, α-enolase, β-actin, and α/β-tubulin, which are all well-known ACPA auto-antigens in the platelets and PDPs (8, 41, 43). In addition, our MS data uncovered a list of citrullinated proteins including numerous cytoskeletal and cell-motility proteins such as myosin-9, caveolae-associated protein 2, tubulin, and actin. Platelets are highly specialized cells and their activation involves a series of rapid rearrangements in the cytoskeleton. In this context, we hypothesized that the citrullination may modify the protein’s functional properties regulating their actin dynamics, similar to the functions of other PTMs present in the actin cytoskeleton like acetylation, phosphorylation, tyrosination and polyglutamylation (22, 24, 44). Furthermore, upon stimulation by various dysregulated molecules in the synovium and circulation, platelet could quick release immunostimulatory microparticals and intracellular citrullinated proteins in their environment, the exposed citrullinated proteins recognition by ACPA leading to formation of inflammatory immune complexes. Thus, activated platelet, immunostimulatory microparticals, and inflammatory immune complexes perpetuate pathogenic mechanisms in RA disease (**Figure 5**).

Intriguingly, we detected citrullination on some cytokines in PDPs, such as PF-4 and TGFβ-1. Previous studies have found that citrullination of cytokines could regulate their biochemical functions. For example, citrullinated chemokines CXCL10, CXCL11, CXCL8 and CXCL12 have reduced chemoattracting capacity, and their ability to signal through the chemokine receptors (45–47). Thus, it is important to underscore that the citrullination process should no longer be considered as a rare modification, because a large number of citrullinated proteins are being identified in RA (8, 10, 34) and in many other diseases as well. Our proteomic analysis of platelets and PDPs has shown the presence of abundant citrullinated proteins/peptides that overlap with ACPA autoantigens. Citrullination of proteins, even among the important targets of autoantibodies ACPA, is a physiological event, not specific for RA, although the production of ACPA is more specific for RA.

Citrullination of TSP-1, β-actin, and PF4 led to exposure of epitopes recognized by ACPA may facilitate our understanding of the role of ACPA in RA development by providing evidences for previous observations involving platelet activation by ACPA in RA (20), the elevated levels of PDPs associated with disease activity (16, 48), and possibly the increased risk of cardiovascular events observed in RA patients (49). Although ACPA and tested platelet used in this study are non-paired sample, we speculate in matched samples, ACPA could reactive with self-generation platelet derived citrullination protein and lead to self-platelet activation, since both citrullinated protein and ACPA are heterogeneity, PTM as citrullinated modification could break of autoreactive B cell tolerance (50).

This study is the first comprehensive description of the platelet citrullination and expands our understanding of the scope of citrullination in platelets. We propose PAD4 enzyme could be responsible for eliciting protein citrullination in human platelets and thus suitable as a target protein for future drug development. In addition, our results may help for better understanding of the origin and consequences of citrullination processes as well as the citrullination-associated autoimmune mechanisms involved in RA.

## Data availability statement

The mass spectrometry proteomics data was deposited in the ProteomeXchange with a dataset identifier PXD037743.

## Ethics statement

The study was approved by the local ethics committee of Union Hospital, Tongji Medical College (No. [2020] IEC-J(130)). The patients/participants provided their written informed consent to participate in this study.

## Author contributions

All authors were involved in drafting the article or revising it critically for important intellectual content, and all authors approved the final version to be published. HG had full access to all of the data in the study and take responsibility for the integrity of the data and the accuracy of the data analysis. All authors contributed to the article and approved the submitted version.
