# Supplementary material for: Platelets derived citrullinated proteins and microparticles are potential autoantibodies ACPA targets in RA patients
Source: Front Immunol. 2023 Jan 24;14:1084283. doi: 10.3389/fimmu.2023.1084283 (PMC9902922; doi:10.3389/fimmu.2023.1084283)
Supplement: Supplementary file 1 [file Table_1.docx]

**Table S1. Primers used for amplification of PAD isoenzymes**

| **Gene** | **Primer** | **Size (bp)** |
| --- | --- | --- |
| PAD 1 | Forward ACCCACAGCTGGCTGATG | 361 |
|  | Reverse CAGTGTCTGTGAAGAGGGT |  |
| PAD 2 | Forward CAAGGAAGATCTCAAGGACA | 814 |
|  | Reverse CTTGGTCATCCTCCGA |  |
| PAD 3 | Forward CTGTGAGGGAAGGCAGG | 314 |
|  | Reverse CATGCCTATAGGCCTCACAC |  |
| PAD 4 | Forward GGACTGCGAGGATGATGAAGTG | 508 |
|  | Reverse CTTGCACTTGGCTTTCATGGCCAGAG |  |
| PAD 6 | Forward ACATACTATGGGCCCAACGAG | 462 |
|  | Reverse CTCCTTCAAGTGGTTCCCGA |  |
| β-actin | Forward ACTCCATCATGAAGTGTGACG | 239 |
|  | Reverse CATACTCCTGCTTGCTGATCC |  |
