# Supplementary material for: Platelets derived citrullinated proteins and microparticles are potential autoantibodies ACPA targets in RA patients
Source: Front Immunol. 2023 Jan 24;14:1084283. doi: 10.3389/fimmu.2023.1084283 (PMC9902922; doi:10.3389/fimmu.2023.1084283)
Supplement: Supplementary file 4 [file DataSheet_1.docx]

Figure S1


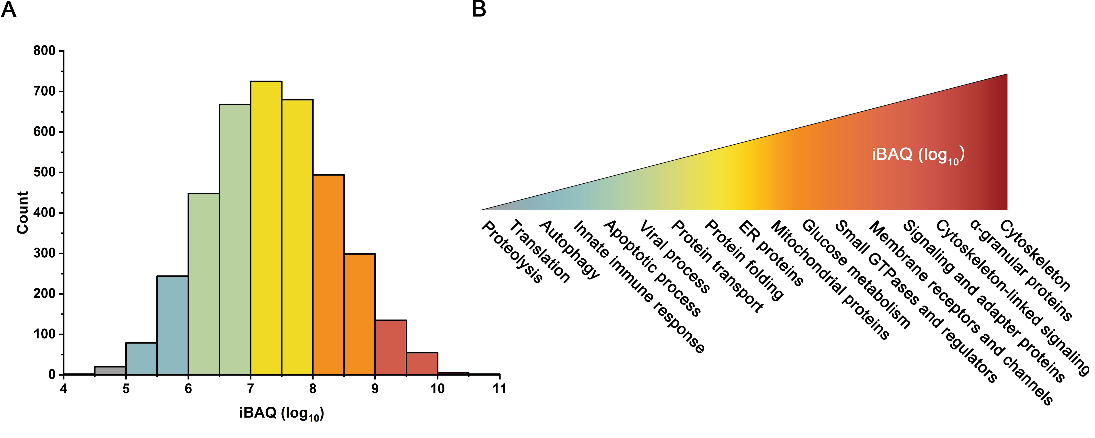


**Figure S1 (A)** Normalized abundance distribution of all identified 3200 proteins in platelets, expressed in log10 values of absolute iBAQ intensities. **(B)** KEGG Gene ontology analysis, showing the pathway enrichment in all proteins measured in platelets.
